# Safety Evaluation of Oral Toxicity of *Carica papaya* Linn. Leaves: A Subchronic Toxicity Study in Sprague Dawley Rats

**DOI:** 10.1155/2014/741470

**Published:** 2014-10-29

**Authors:** Zakiah Ismail, Siti Zaleha Halim, Noor Rain Abdullah, Adlin Afzan, Badrul Amini Abdul Rashid, Ibrahim Jantan

**Affiliations:** ^1^Herbal Medicine Research Center, Institute for Medical Research, Jalan Pahang, 50588 Kuala Lumpur, Malaysia; ^2^Infectious Disease Research Center, Institute for Medical Research, Jalan Pahang, 50588 Kuala Lumpur, Malaysia; ^3^Drug and Herbal Research Center, Faculty of Pharmacy, Universiti Kebangsaan Malaysia, Jalan Raja Muda Abdul Aziz, 50300 Kuala Lumpur, Malaysia

## Abstract

The subchronic toxicity effect of the leaf extract of *Carica papaya* Linn. in Sprague Dawley (SD) rats was investigated in this study. The extract was prepared by dissolving the freeze dried extract of the leaves in distilled water and was administered orally to SD rats (consisted of 10 rats/sex/group) at 0 (control), 0.01, 0.14, and 2 g/kg body weight (BW) for 13 weeks. General observation, mortality, and food and water intake were monitored throughout the experimental period. Hematological and biochemical parameters, relative organ weights, and histopathological changes were evaluated. The study showed that leaf extract when administered for 13 weeks did not cause any mortality and abnormalities of behavior or changes in body weight as well as food and water intake. There were no significant differences observed in hematology parameters between treatment and control groups; however significant differences were seen in biochemistry values, for example, LDH, creatinine, total protein, and albumin. However, these changes were not associated with histopathological changes. In conclusion, the results suggested that daily oral administration of rats with *C. papaya* leaf extract for 13 weeks at a dose up to fourteen times the levels employed in traditional medicine practice did not cause any significant toxic effect.

## 1. Introduction

*Carica papaya* Linn. (family: Caricaceae) is primarily cultivated for its fruit and the young leaves are consumed as a vegetable by the Malay community in Malaysia. Different parts of the plant have been used in traditional medicine to treat various diseases. The juice of the green leaves is consumed as beverage to treat malarial fever [1]. The leaves are also used to treat digestive disorders and other disturbances of the gastrointestinal tract. The fruit is used to treat high fever, cough, and anorexia and the seed has been used to treat digestive disorders, to improve protein digestion, and to expel intestinal worms [2]. The root is used to treat urinary disorders while the bark is used to treat toothache [3].

Many studies have been conducted to evaluate the biological activities of various parts of* C. papaya*. The fruit and seed of* C. papaya* have showed bacteriostatic activity against several enteropathogens in human [4]. It was reported that experimentally induced diabetic rats showed significant wound healing after treatment with the aqueous extract of the fruit of* C. papaya* [5]. The aqueous extract of the leaves of the plant showed significant protective effect against alcohol induced oxidative damage to the gastric mucosa in rats [6], and it was effective in healing induced wound in rats [7]. The aqueous extract of the leaves also exhibited the ability to inhibit tumor cell lines [8] and this activity was potentiated with phenolic compounds [9, 10]. Xanthine oxidase was found to be present in the leaf extract and thus it has potential to work as an anti-gout agent [11]. The crude ethyl acetate extract of* C. papaya* leaves showed high antiplasmodial activity against* Plasmodium falciparum* and* P. falciparum*-resistant strains [12]. The ethanol extract of* C. papaya* leaves showed potential as a source of drugs against urinary tract microbes [13].

Phytochemical studies on the leaves of* C. papaya* showed the presence of various compounds including piperidine alkaloids such as carpaine, pseudocarpaine, and dehydrocarpaines I and II [14–17]. The alkaloids have been shown to be teratogenic to livestock as they were found to inhibit the fetal movement [18] and exhibited antiamoebic activity [19]. Ekong et al. [20] also reported that the aqueous extract of* C. papaya* leaves given to pregnant rats showed some defects in fetus. The leaves can be considered to possess some nutritional value as they were found to contain multinutrients and iron [21]. Recently we identified carpaine, malic acid, quinic acid, six malic acid derivatives, and four flavonol glycosides in the leaf extract of the plant [22].

Toxicity evaluation of* C. papaya* leaves becomes more important as they are not only consumed widely as food but also prepared and used as a traditional medicine. Acute toxicity study which involved a single dose administration of* C. papaya* leaf juice and is followed by fourteen days' observation in rats up to 2 g/kg BW showed dehydration as demonstrated by an increase in red cell mass [23]. We have also carried out a repeated dose 28-day oral toxicity study of the leaf extract in rats (subacute toxicity study) and the results indicated that the plant extract did not cause mortality, there were no treatment-related changes, and all organs did not reveal morphological alterations [22]. We also have carried out a clinical trial to investigate the platelet elevating property in dengue patients after having received* C. papaya* leaf juices for 3 consecutive days [24].

The present study, a subchronic toxicity evaluation of* C. papaya* leaf extract, was carried out to determine long term consumption effect after repeated doses of* C. papaya* leaf extract in a thirteen-week oral toxicity study.

## 2. Materials and Methods 

### 2.1. Plant Material

The fresh leaves of* Carica papaya* L. “Sekaki” were purchased by the Malaysian Agricultural Research and Development Institute (MARDI). A representative sample of this plant was authenticated at the Forest Research Institute Malaysia (FRIM), Kepong, with voucher specimen number 007/10. Leaves were collected and washed under running tap water and then cut into small pieces and juice was extracted using a juicer (Panasonic, Shah Alam, Malaysia). The resulting juice, without addition of water, was then poured into a glass container and left frozen in a freezer. The juice was lyophilized resulting in a dark green powder (2.6% w/w yield). Phytochemical analysis had been carried out for chemical fingerprinting [22]. The doses used in the toxicity study were calculated based on the body weight (BW) of the rats. The test samples were prepared by dissolving the powder in distilled water to obtain concentrations of 0.01, 0.14, and 2 g/kg BW.

### 2.2. Animals

Male and female Sprague Dawley (SD) rats aged between six and seven weeks and weighed between 90 and 100 g were used in this study. The SD rats were obtained from the Laboratory Animal Resource Unit, Medical Resource Research Center, Institute for Medical Research (IMR), Kuala Lumpur. The use of laboratory animals and the study design were approved by the Institutional Animal Care and Used Committee (IACUC) (ACUC number ACUC/KKM 02 (1/2009)). The Guidelines of Handling of Laboratory Animals by the Ministry of Health Malaysia were followed throughout the experiments [25].

The animals were housed individually in a stainless-steel wire-mesh cage with size 6H × 11D × 16W cm and maintained at room temperature (27 ± 2°C) with humidity of 65.85 ± 6.76% and with 12 h alternate artificial and natural light and dark cycle. Room temperature and relative humidity were recorded daily using a temperature datalogger (TempRH Datalogger BG-DL-01/01B). Each animal was identified by a cage card. They were fed with a pellet diet with Zeigler Rodent NIH-31 irradiated auto wafer feeds (Zeigler Bros, Pennsylvania, USA) and given an unlimited supply of reverse osmosis water. The animals were acclimatized to laboratory conditions for seven days prior to the experiments.

### 2.3. Experimental Design

The subchronic toxicity study was carried out according to the OECD Guidelines for the “Repeated Dose 90-day Oral Toxicity Study in Rodents,” no. 408 [26] with some modifications. The modifications were in the temperature and humidity of the room. Forty female and 40 male rats were randomly assigned into four groups which were made up of one control group and three treatment groups (*n* = 10 rats/sex/group). The treatment group received lyophilized* C. papaya* leaf juice diluted in water to the necessary dosage while the control group received water only.

### 2.4. Selection of Doses and Oral Administration of the Extracts

Dose selection was based on the acute toxicity and subacute toxicity studies carried out previously, where the highest dose of 2 g/kg BW did not exhibit any acute effects on the rats (NOAEL) [23]. Therefore, a dose of 2 g/kg BW was selected for the highest dose in this study. The medium (0.14 g/kg BW) and low dose (0.01 g/kg BW) levels were selected based on the local traditional preparation of* C. papaya* juice for treatment of fever in humans. The* C. papaya* leaf juice at different concentrations was administered orally using intubation needle on a daily basis for 13 weeks. The rats were weighed weekly and the amount of* C. papaya* leaf juice to be given was recalculated based on the new body weight to ensure a constant dose volume per kg BW at all times. Control rats were administered the same volume of drinking water as the amount given to the test groups.

### 2.5. Parameters Measured during the Study

#### 2.5.1. General Observation and Mortality

General observations were carried out twice daily for mortality, moribund and ill health, or reaction to treatment. These observations include changes in skin, fur, eyes, mucus membranes, behavior pattern, tremors, salivation, diarrhea, sleep, and coma. The observation was carried out according to the Guidance Document on the Recognition, Assessment and Use of Clinical Signs as Humane Endpoints for Experimental Animals Used in Safety Evaluation [27].

#### 2.5.2. Body Weight and Food and Water Consumption

Individual rat was weighed before the commencement of the experiment and then weighed once on day 7 of every week. Final body weights were recorded prior to the scheduled necropsy. Each cage was supplied with calculated amounts of food and water. The amounts of left over food and water were measured weekly and the differences were regarded as food (g/rat/week) and water consumption (mL/rat/week).

#### 2.5.3. Hematological and Biochemical Analysis

The rats were fasted overnight by removing all food from the cages but were allowed access to water ad libitum before blood was collected. Rats were anaesthetized with light ether and blood samples were collected via direct heart puncture and put into two types of tubes, one with anticoagulant (EDTA) and the other without any additives. After withdrawing blood from the rats, they were sacrificed with an overdose of ether. The anticoagulated blood samples (EDTA) were analyzed immediately for hematology parameters using Hematology Analyzer (Medonic CA 620 VET, Stockholm, Sweden). These parameters include total and differential leukocyte count (WBC), erythrocyte counts (RBC), hemoglobin concentration (HGB), hematocrit (HCT), mean cell volume (MCV), mean corpuscular hemoglobin (MCH), mean corpuscular hemoglobin concentration (MCHC), and platelet counts (PLT). The blood without any additives was used in the biochemistries and the parameters which include serum total protein, albumin, alkaline phosphatase (ALP), aspartate aminotransferase (AST), alanine aminotransferase (ALT) urea, creatinine, uric acid, creatinine kinase (CK), lactate dehydrogenase (LDH), *α*-hydroxybutyrate dehydrogenase (HBDH), cholesterol, triglycerides, and glucose were determined. The tests were done using the biochemistry analyser (Vitalab Selecta, E-series, Netherlands).

#### 2.5.4. Gross Findings and Organ Weights

Complete postmortem examinations were carried out on all animals. The rats were dissected, and their internal organs were carefully examined for any pathological changes. Subsequently the organs were removed and weighed. The organs were the lungs, heart, liver, stomach, spleen, gastrointestinal, kidneys, testes, and adrenals. The relative organ weight (ROW) of each organ was calculated using the following equation:

(1) ROW=[Absolute  organ  weight(g)Body  weight  of  rat  on⁡  sacrifice  day(g)] ×100.

The organs were preserved in 10% buffered formalin for subsequent histopathological examination.

#### 2.5.5. Histopathology

For histopathological examination, a representative tissue or the whole organ (depending on the size and weight) was taken and processed further to make slides using the standard procedure for histology slides and was stained with Hematoxylin and Eosin. The slides were examined using a light microscope (Olympus BX 51, Tokyo, Japan).

### 2.6. Statistical Analysis

The data were analyzed using SPSS program, version 14. The one-way analysis of variance (ANOVA) was used to test for significant differences between the experimental groups and was followed by Tukey's HSD for multiple comparisons [28, 29]. Nonparametric test methods were used when the distribution of certain variable(s) differed from normal. The nonparametric methods employed were the Kruskal-Wallis test for pairwise comparison. Results with *P* < 0.05 were considered statistically significant. The results were expressed as mean value (*x*) and standard deviation (SD) for each variable measured.

## 3. Results

### 3.1. Survival and General Observation

There was no treatment related death reported in any of the experiment groups as well as in the control group. Neither physical nor behavioral changes were observed in any of the groups throughout the study period of 13 weeks.

### 3.2. Body Weight Changes and Food and Water Consumption

**Figure 1:**
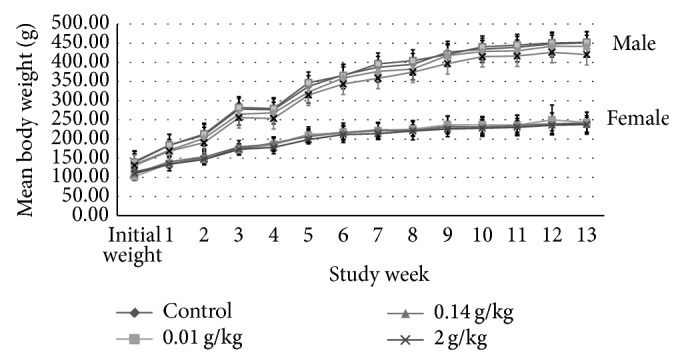
Mean body weight (g) of male and female rats administered daily after subchronic treatment orally for 13 weeks with* C. papaya* leaf extract in SD rats. Values are expressed as mean ± standard deviation (*n* = 10/group).

**Figure 2:**
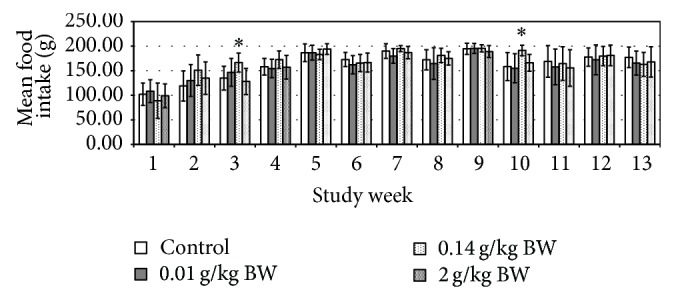
Mean food intake (g) of rats administered daily after subchronic treatment orally for 13 weeks with* C. papaya* leaf extract in male SD rats. Values are expressed as mean ± standard deviation (*n* = 10/group). (^*^) *P* < 0.05 significant value.

**Figure 3:**
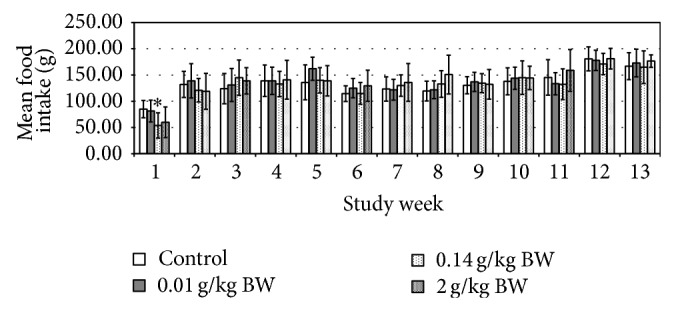
Mean food intake (g) of rats administered daily after subchronic treatment orally for 13 weeks with* C. papaya* leaf extract in female SD rats. Values are expressed as mean ± standard deviation (*n* = 10/group). (^*^) *P* < 0.05 significant value.

**Figure 4:**
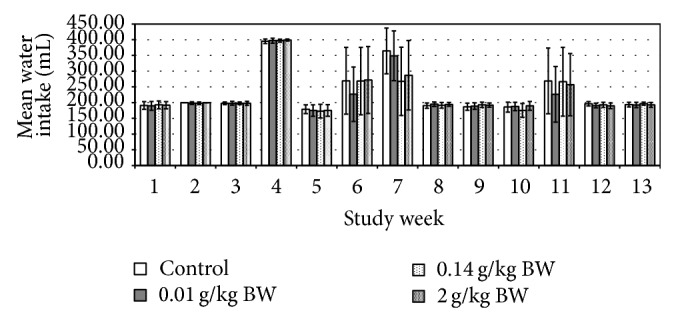
Mean water intake (mL) of rats administered daily after subchronic treatment orally for 13 weeks with* C. papaya* leaf extract in female SD rats. Values are expressed as mean ± standard deviation (*n* = 10/group).

**Figure 5:**
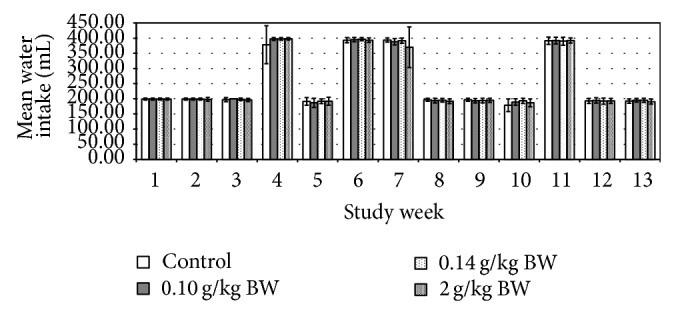
Mean water intake (mL) of rats administered daily after subchronic treatment orally for 13 weeks with* C. papaya* leaf extract in male SD rats. Values are expressed as mean ± standard deviation (*n* = 10/group).

The initial (day 0) body weights of the female rats were 436.00 ± 2.33 g, whereas for the male rats were 545.00 ± 1.14 g. Their body weights were gradually increased as noted in weekly measurements and presented in Figure 1. No significant difference in body weight changes was noted between the control group and any of the treated groups at any time of the 13-week period. The male test group receiving medium dose in week 3 and week 10 showed significant increase in food consumption (*P* = 0.008, *P* = 0.012, resp.) (Figure 2). While the female test group which received medium dose showed significant decrease (*P* = 0.010) in food consumption during week 1 (Figure 3). However, there is no significant difference in the amount of water consumed in all various groups, including the control, throughout the study period (Figures 4 and 5, resp.).

### 3.3. Hematology and Biochemistry

The hematological and the biochemistry values are presented in Tables 1 and 2, respectively. There were no significant changes seen in all the hematological parameters amongst the different treatment groups as well as the control group for both male and female rats. However, for biochemistry values, some significant changes were seen. In the male rats treated with medium dose, they showed significant increase in albumin and LDH level (*P* = 0.001 and *P* = 0.005, resp.). While rats were treated with high dose, a significant decrease in creatinine and a significant increase in LDH (*P* = 0.001 and *P* = 0.020, resp.) were seen. The female rats treated with medium and high doses showed a significant increase in total protein (*P* = 0.005 and *P* = 0.001, resp.). The albumin values for rats treated with medium and high doses were significantly decreased with *P* = 0.001 and *P* = 0.001, respectively. The creatinine value was significantly decreased in rats treated with high dose (*P* = 0.005). The glucose value was significantly increased (*P* = 0.031) in rats treated with low dose.

### 3.4. Organs Weight Changes

As mentioned above in the methodology, the body weight and organ weight of individual rats were recorded at necropsy. The relative organ weight (ROW) of each organ was calculated. The data showed that there were no significant differences in all the relative organs weights as compared to the control rats (Table 3).

### 3.5. Histopathology

**Figure 6:**
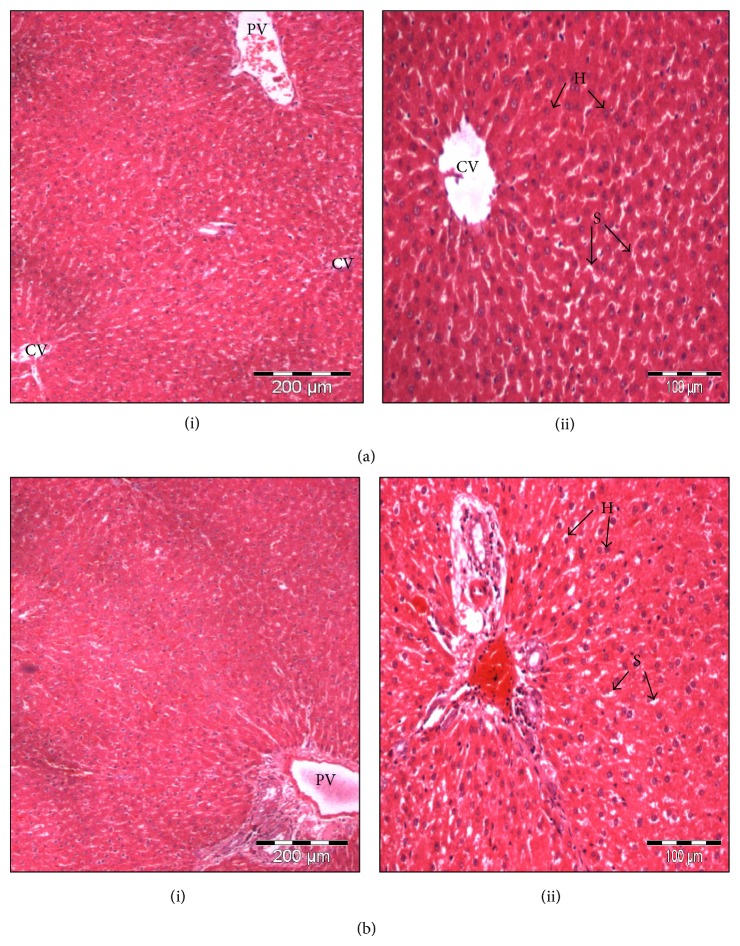
Histological structure of liver from control (a) medium 0.14 g/kg BW (b) group of male SD rat showing the Central vein (CV), hepatocyte (H), the sinusoid (S) and the portal vein (PV). The structure showed there were normal as compared to the control. (i) 4x, (ii) 40x, (H&E Staining).

Histological examination revealed no significant changes in all the organs examined including liver as compared to the control groups, some parts of the picture from the histopathology in Figure 6. They were normal compared to the control group.

## 4. Discussion

Safety of long term use or consumption of medicinal plants is becoming important as most of this preparation will be for general health and will be used for long term duration. In order to elucidate such information, a proper toxicological evaluation is carried out in various experimental animal models to predict toxicity and to select a “safe” dose for human use. Subsequent to that, the safety data on human subject should be conducted through the various phases of clinical trial.

General toxicity studies will normally include acute toxicity, subacute toxicity, and chronic or subchronic toxicity studies. As mentioned earlier, both acute and subacute toxicity studies had been conducted on* C. papaya* leaves juice and both showed no significant findings that lead to meaningful interpretation of toxic effect. The acute toxicity study of* C. papaya* leaf juice in rats showed dehydration as demonstrated by an increase in red cell mass [23]. We have also carried out a repeated dose 28-day oral toxicity study of a similar leaf extract in rats and the results indicated that the plant extract did not cause mortality, there were no treatment-related changes, and all organs did not reveal morphological alterations [22]. However the biochemistry values (total protein, AST, ALT, and ALP) revealed some changes although they are non-dose dependent. Thus, a subchronic toxicity evaluation of* C. papaya* leaf extract is necessary to confirm the finding especially when the dosing is given for a longer period in this thirteen-week oral toxicity study. The same aqueous juice extract of* C. papaya* leaves was also used in this study and such formulation was the form that traditionally had been consumed [30].

From the above presented result, it was showed that daily administration of the* C. papaya* leaves juice for 13 weeks at the chosen doses did not show any changes in the general behaviors of treated rats, all gained weight normally corresponding to the food and water intake. It was noted that during weeks 4, 6, 7, and 11 the water intake was increased in both female and male rats in all groups. However there were no significant differences between groups as compared to the control group. These increases of water intake did not show any change or directly proportional to body weight and food intake; hence, it was more of an accidental finding and was not related to the administration of* C*.* papaya* leaf juice. As evidenced by the absence of toxic symptoms, there were no changes in food intake and body weight in those weeks. As seen in the previous studies, there were no hematological changes seen in all the parameters measured including the platelets and hematocrits.

The biochemistry values showed some significant changes in the liver enzymes as in the previous 2 studies; however there is no dose related pattern was observed. At the current subchronic study, all the liver enzymes (AST, ALT, and ALP) except LDH were not significantly different from the control group in both male and female rats. The LDH was seen to be increasing significantly but only in male group with medium (with increased albumin) and high doses (with decreased level of creatinine), while in female group, such finding was not noted. Previous researchers describe possible liver dysfunction when such studies were conducted for the herbal product/plant. Everds [31] reported that an increase in albumin level maybe associated to abnormal liver function or dehydration state. As discussed by Ramaiah [32], abnormality in AST, ALT, and ALP levels is more specific for liver cell injury. Garba and Ubom [33] and Kotoh et al. [34] reported that LDH also can be used as indicator in liver cell injures as the enzymes are released from the injured hepatocytes although the specificity to indicate liver disease is lower compared with AST, ALT and ALP. This is due to the fact that serum LDH in liver more rapidly declines and also might indicate the presence of red cell hemolysis rather than liver cell injuries. The other authors, Preus et al. [35] and Oloyede and Sunmonu [36], mention the LDH as a useful indicator for cardiac damage. In the current study, the finding is most likely to indicate liver damaged rather than red cell hemolysis or cardiac damaged although the other specific liver enzymes AST, ALT, and ALP were not significantly different, whereas in the female group, the medium and high group showed significant increase in total protein, *P* = 0.005 and *P* = 0.001, respectively, but their albumin values were significantly decreased with *P* = 0.001 and *P* = 0.001, respectively. This could be due to the fact that other protein fractions other than albumin might be increased and thus need to be further investigated.

As the rats are aging and their size increases, their physical activities reduced and hence this could be the explanation for the significant reduction of creatinine level in both male and female rats treated with the highest dose. This is in reference to reports by Omer [37], Attia and Nasr [38] which described that creatinine values were affected by the variation of body weight or physical activity. Creatinine and urea are waste products of protein excreted from kidney and are indicators for kidney damage. Thus this reduction of creatinine indicates that there was no potential kidney effect and this was supported by the urea value which remained normal as compared to the control group.

Tarkang et al. [39] also conducted study on aqueous as well as ethanol extracts of the leaves of* C. papaya* for 28 and 90 days. The finding showed no abnormalities in liver enzymes and renal biochemistries in rat after administration of* C. papaya* leaf extract for 28 and 90 days. Only ethanol extract showed some changes in the liver and renal toxicity at the dose 1 g/kg BW. This different finding could be due to the fact that different extract was used in the study. It was air-dried and extracted either with water or ethanol, while juice extracted from the fresh leaves was used in this study.

## 5. Conclusions

In conclusion, the administration of rats with low, medium, and high doses of fresh juice of* C. papaya* leaf extract for 13 weeks did not cause any changes in body weight, food intake, and water level. There were also no significant differences observed in hematology parameters between treatment and control groups. There were significant differences in biochemistry values, such as the LDH, creatinine, total protein, and albumin. However, these changes were not associated with histopathological changes and were not dose dependent. Such finding on possible liver dysfunction needs to be confirmed with proper hepatotoxicity study protocol to elucidate extension of the toxic effect if any. The oral dose of* C. papaya* leaf extract was more than 2 g/kg BW and no observed adverse effect level (NOAEL) of the extract for both female and male rats was 2 g/kg BW per day for 13 weeks extracts on Sprague Dawley rats for the present study.

## Figures and Tables

**Table 1:** Hematological values of control group and rats treated with *C. papaya* leaf extract measured during the subchronic toxicity study.

Male rats	Control	0.01 g/kg BW	0.14 g/kg BW	2 g/kg BW
WBC (10³/*μ*L)	4.74 ± 2.04	6.01 ± 2.84	3.69 ± 1.55	6.24 ± 2.73
RBC (10^6^×*μ*L)	7.89 ± 0.42	7.96 ± 0.40	7.88 ± 0.51	8.03 ± 0.37
HGB (g/dL)	16.30 ± 0.49	16.13 ± 0.72	16.27 ± 0.81	16.34 ± 0.73
HCT (%)	40.08 ± 1.42	39.83 ± 1.97	40.11 ± 1.87	39.97 ± 1.86
MCV (fL)	50.84 ± 1.69	49.98 ± 1.32	50.94 ± 1.74	49.79 ± 1.55
MCH (pg)	20.69 ± 0.74	20.24 ± 0.53	20.67 ± 0.54	20.38 ± 0.56
MCHC (g/dL)	40.70 ± 0.50	40.51 ± 0.42	40.58 ± 0.49	40.93 ± 0.50
PLT (10³/*μ*L)	637.67 ± 100.04	697.88 ± 69.91	563.44 ± 209.03	718.67 ± 111.74

Female rats	Control	0.01 g/kg BW	0.14 g/kg BW	2 g/kg BW

WBC (10³/*μ*L)	2.13 ± 0.98	1.71 ± 0.97	1.65 ± 1.25	3.40 ± 2.41
RBC (10^6^×*μ*L)	7.22 ± 0.45	7.05 ± 0.21	6.89 ± 0.51	6.85 ± 0.68
HGB (g/dL)	15.57 ± 0.79	15.16 ± 0.38	14.61 ± 1.09	14.38 ± 1.29
HCT (%)	37.57 ± 1.97	36.55 ± 0.98	35.45 ± 2.83	34.70 ± 3.29
MCV (fL)	52.08 ± 1.59	51.87 ± 0.97	51.45 ± 1.04	50.70 ± 1.81
MCH (pg)	21.59 ± 0.72	21.52 ± 0.46	21.23 ± 0.42	21.04 ± 0.67
MCHC (g/dL)	41.47 ± 0.62	41.49 ± 0.48	41.29 ± 0.60	41.54 ± 0.45
PLT (10³/*μ*L)	708.40 ± 117.74	681.70 ± 56.09	464.50 ± 272.37	535.00 ± 290.60

Values are expressed as mean ± standard deviation (*n* = 10/group). WBC: white blood cells, RBC: red blood cells, HGB: Hemoglobin, HCT: hematocrit, MCV: mean corpuscular volume, MCH: mean cell hemoglobin, MCHC: mean corpuscular hemoglobin concentration, and PLT: platelet. ^*∗*^ *P* value less than 0.05 (*P* < 0.05), significant value.

**Table 2:** Biochemistry values of control group and rats treated with *C. papaya* leaf extract measured during the subchronic toxicity study.

Male rats	Control	0.01 g/kg BW	0.14 g/kg BW	2 g/kg BW
Liver profile				
Total protein (g/L)	57.22 ± 2.44	59.13 ± 4.76	59.11 ± 5.73	60.89 ± 8.18
Albumin (g/L)	27.51 ± 2.90	28.60 ± 2.70	32.32 ± 2.19^*^	29.67 ± 1.75
ALP (U/L)	147.38 ± 33.20	173.13 ± 61.47	192.89 ± 60.99	218.22 ± 98.43
AST (U/L)	186.38 ± 28.16	166.63 ± 41.81	169.67 ± 37.53	158.22 ± 39.52
ALT (U/L)	49.50 ± 8.62	46.75 ± 7.17	61.22 ± 23.63	60.78 ± 29.82
Renal profile				
Urea (mmol/L)	6.75 ± 0.51	6.14 ± 1.12	6.73 ± 0.68	5.73 ± 1.22
Creatinine (*μ*mol/L)	56.25 ± 8.99	51.75 ± 7.13	49.89 ± 8.10	40.33 ± 16.38^*^
Uric acid (*μ*mol/L)	117.23 ± 50.93	81.53 ± 25.60	161.33 ± 98.28	95.80 ± 43.12
Cardiac profile				
CK (U/L)	1408.250 ± 534.91	1187.25 ± 208.64	1589.11 ± 588.53	1448.78 ± 478.84
LDH (U/L)	2014.67 ± 500.68	2187.38 ± 875.63	2706.00 ± 645.85	2952.78 ± 259.86^*^
HBDH (U/L)	648.33 ± 119.92	612.63 ± 199.95	648.00 ± 161.63	589.00 ± 106.74
Lipid profile				
Cholestrol (mmol/L)	1.39 ± 1.18	1.12 ± 0.53	1.21 ± 0.21	1.27 ± 0.28
Triglycerides (mmol/L)	0.97 ± 0.51	0.80 ± 0.30	1.27 ± 0.22	1.26 ± 0.31
Glucose (mmol/L)	7.97 ± 2.68	6.96 ± 2.01	10.31 ± 5.47	7.68 ± 2.84

Female rats	Control	0.01 g/kg BW	0.14 g/kg BW	2 g/kg BW

Liver profile				
Total protein (g/L)	71.80 ± 4.10	71.33 ± 3.84	89.00 ± 18.18^*^	87.33 ± 6.93^*^
Albumin (g/L)	44.84 ± 2.54	44.43 ± 2.26	34.92 ± 5.08^*^	32.58 ± 2.22^*^
ALP (U/L)	119.20 ± 53.88	169.78 ± 80.00	70.30 ± 51.41	95.78 ± 40.09
AST (U/L)	274.20 ± 47.48	276.78 ± 83.09	268.80 ± 147.28	215.89 ± 72.40
ALT (U/L)	70.80 ± 18.50	85.89 ± 21.74	63.10 ± 33.51	52.33 ± 25.07
Renal profile				
Urea (mmol/L)	9.21 ± 1.45	8.78 ± 1.00	7.51 ± 2.11	7.38 ± 2.13
Creatinine (*μ*mol/L)	78.56 ± 10.76	83.56 ± 14.04	69.70 ± 14.09	57.67 ± 8.20^*^
Uric acid (*μ*mol/L)	277.52 ± 119.04	440.73 ± 185.39	359.33 ± 195.94	247.36 ± 97.58
Cardiac profile				
CK (U/L)	1675.89 ± 364.79	1514.75 ± 517.94	1530.30 ± 671.40	1739.11 ± 1163.74
LDH (U/L)	1481.50 ± 871.06	2265.50 ± 852.88	1561.70 ± 593.89	1683.11 ± 668.59
HBDH (U/L)	1027.78 ± 302.57	867.00 ± 171.88	888.50 ± 123.73	817.25 ± 148.18
Lipid profile				
Cholestrol (mmol/L)	1.62 ± 0.23	1.56 ± 0.23	1.79 ± 0.41	1.57 ± 0.29
Triglycerides (mmol/L)	1.31 ± 0.29	1.47 ± 0.36	1.43 ± 0.26	1.48 ± 0.15
Glucose (mmol/L)	8.88 ± 5.92	15.17 ± 5.62^*^	9.24 ± 3.04	6.85 ± 1.73

Values are expressed as mean ± standard deviation (*n* = 10/group). ALP: alkaline phosphatase, AST: aspartate aminotransferase, ALT: alanine aminotransferase, CK: creatinine kinase, LDH: lactate dehydrogenase, and HBDH: *α*-hydroxybutyrate dehydrogenase. ^*∗*^ *P* value less than 0.05 (*P* < 0.05), significant value.

**Table 3:** Organ weight values of control group and rats treated with *C. papaya* leaf extract measured during the subchronic toxicity study. The relative organ weight per 100 g body weight recorded at the end of the study.

Organ	Control	0.01 g/kg BW	0.14 g/kg BW	2 g/kg BW
Male rats				
Lung	0.31 ± 0.03	0.36 ± 0.09	0.37 ± 0.07	0.35 ± 0.08
Heart	0.27 ± 0.02	0.29 ± 0.05	0.27 ± 0.02	0.27 ± 0.03
Liver	2.87 ± 0.14	3.05 ± 0.66	2.80 ± 0.37	2.82 ± 0.27
Stomach	0.38 ± 0.03	0.38 ± 0.05	0.39 ± 0.06	0.37 ± 0.05
Spleen	0.13 ± 0.02	0.15 ± 0.01	0.15 ± 0.01	0.15 ± 0.03
GIT	0.34 ± 0.13	0.36 ± 0.08	0.28 ± 0.08	0.32 ± 0.09
Kidney (left)	0.31 ± 0.03	0.30 ± 0.06	0.29 ± 0.02	0.29 ± 0.03
Kidney (right)	0.32 ± 0.03	0.29 ± 0.05	0.29 ± 0.02	0.29 ± 0.03
Testis (left)	0.35 ± 0.03	0.34 ± 0.04	0.34 ± 0.03	0.35 ± 0.03
Testis (right)	0.35 ± 0.03	0.34 ± 0.04	0.35 ± 0.04	0.34 ± 0.02
Adrenal (left)	0.01 ± 0.00	0.01 ± 0.00	0.01 ± 0.00	0.01 ± 0.00
Adrenal (right)	0.01 ± 0.00	0.01 ± 0.00	0.01 ± 0.00	0.00 ± 0.00
Female rats				
Lung	0.46 ± 0.06	0.45 ± 0.09	0.44 ± 0.07	0.45 ± 0.09
Heart	0.32 ± 0.03	0.31 ± 0.02	0.31 ± 0.02	0.31 ± 0.03
Liver	2.95 ± 0.30	3.02 ± 0.27	2.85 ± 0.19	3.02 ± 0.26
Stomach	0.50 ± 0.07	0.46 ± 0.07	0.51 ± 0.08	0.49 ± 0.08
Spleen	0.17 ± 0.02	0.17 ± 0.02	0.18 ± 0.02	0.18 ± 0.02
GIT	0.45 ± 0.12	0.45 ± 0.18	0.43 ± 0.06	0.39 ± 0.04
Kidney (left)	0.30 ± 0.03	0.29 ± 0.04	0.29 ± 0.03	0.31 ± 0.03
Kidney (right)	0.31 ± 0.03	0.31 ± 0.03	0.30 ± 0.02	0.31 ± 0.02
Ovary (left)	0.02 ± 0.01	0.02 ± 0.00	0.02 ± 0.01	0.04 ± 0.06
Ovary (right)	0.02 ± 0.00	0.02 ± 0.00	0.02 ± 0.01	0.02 ± 0.01
Adrenal (left)	0.01 ± 0.00	0.02 ± 0.00	0.01 ± 0.01	0.01 ± 0.00
Adrenal (right)	0.01 ± 0.01	0.01 ± 0.00	0.01 ± 0.00	0.01 ± 0.01

Values are expressed as mean ± standard deviation (*n* = 10/group).
